# LOICA: Integrating Models with Data for Genetic Network Design Automation

**DOI:** 10.1021/acssynbio.1c00603

**Published:** 2022-05-04

**Authors:** Gonzalo Vidal, Carlos Vidal-Céspedes, Timothy J. Rudge

**Affiliations:** † Institute for Biological and Medical Engineering, Schools of Engineering, Biology, and Medicine, Pontificia Universidad Católica de Chile, Santiago 7820244, Chile; ‡ Interdisciplinary Computing and Complex BioSystems (ICOS) Research Group, School of Computing, Newcastle University, Newcastle upon Tyne NE1 7RU, U.K.

**Keywords:** genetic network, genetic design automation, modeling, characterization, dynamical systems, design abstraction

## Abstract

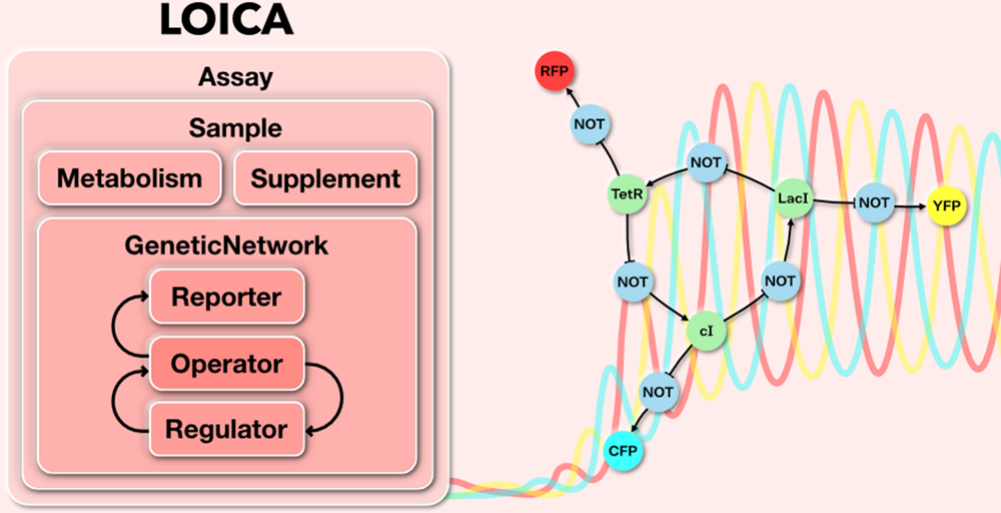

Genetic design automation tools are necessary to expand the scale and complexity of possible synthetic genetic networks. These tools are enabled by abstraction of a hierarchy of standardized components and devices. Abstracted elements must be parametrized from data derived from relevant experiments, and these experiments must be related to the part composition of the abstract components. Here we present Logical Operators for Integrated Cell Algorithms (LOICA), a Python package for designing, modeling, and characterizing genetic networks based on a simple object-oriented design abstraction. LOICA uses classes to represent different biological and experimental components, which generate models through their interactions. These models can be parametrized by direct connection to data contained in Flapjack so that abstracted components of designs can characterize themselves. Models can be simulated using continuous or stochastic methods and the data published and managed using Flapjack. LOICA also outputs SBOL3 descriptions and generates graph representations of genetic network designs.

## Introduction

Synthetic biology is an interdisciplinary field that mixes life sciences and engineering. From this perspective, living systems are objects to engineer, and a rational way to design them is by modifying their genetic code. This can be done by introducing synthetic DNA that encodes a synthetic regulatory network, also known as a genetic network or genetic circuit. The design–build–test–learn (DBTL) cycle is central to engineering disciplines, and each phase requires appropriate tools, standards, and workflows, which are still in development. Synthetic Biology Open Language (SBOL) is an open standard for the representation of *in silico* biological designs that covers the DBTL cycle and has attracted a community of developers that have produced an ecosystem of software tools.^1−4^

Modeling is key to the DBTL cycle and is essential to the design and learn stages since a model states a well-defined hypothesis about the system operation. Abstraction enables the construction and analysis of models based on components, devices, and systems that can be used to compose genetic networks and derive their DNA sequences. It is the basis for genetic design automation (GDA), which can accelerate and automate the genetic network design process by compiling models into DNA sequences. In order for GDA to proceed in a rational way, the abstract elements of genetic networks must be accessible to characterization, allowing parametrization of models of their operation and interactions.

Functional abstraction of DNA sequences as parts such as transcriptional promoters, ribosome binding sites (RBSs), coding sequences (CDSs), terminators, and other elements has enabled the assembly of relatively small genetic networks.^5−7^ However, for large-scale genetic network design, higher-level abstractions are required, as provided by the logic formalism.^8^ In this approach, network compositions are abstracted into genetic logic gates that transition between discrete low and high steady-state gene expression levels according to input signals, either external or internal to the network.^9^ These genetic logic networks can be designed automatically using Cello,^8^ in an analogous way to electronic circuits, on the basis of the required discrete logical truth table, but this specification requires knowledge of the domain-specific programming language Verilog.^10^

Despite the discrete logical design formalism, these genetic networks are dynamical systems and can have autonomous, continuous, non-steady-state dynamics, displaying complex and rich behaviors from bistability to oscillations and even chaos.^4−6^ Furthermore, typical operating conditions for engineered networks such as colonies, bioreactors, and microbiomes are time-varying, which can lead to complex behaviors from even simple genetic networks.^11^

To design genetic networks, we therefore require kinetic gene expression data generated at the test phase. These data must be integrated with models to enable characterization of abstracted parts, devices, and systems as well as metadata, including the DNA part composition and sequence, to enable automated design. Thus, there is a need for software design tools that integrate abstract network designs, dynamical models, kinetic gene expression data, DNA part composition, and sequence *via* common exchange standards in a user-friendly and accessible fashion.

Logical Operators for Integrated Cell Algorithms (LOICA) is a tool for the design, modeling, and parametrization of synthetic genetic networks. In contrast to existing genetic network design and modeling tools,^4^ rather than composing individual genetic parts, LOICA provides a high-level design abstraction that simplifies the design process by representing networks as combinations of components accessible to parametrization. This parametrization of genetic network models is enabled by direct connection to experimental data *via* Flapjack, which also provides a platform for publishing and sharing simulation results. Furthermore, while LOICA abstracts genetic networks at a higher level, designs can be represented using the latest SBOL3 standard. LOICA is a Python package allowing programmatic design, simulation, parametrization, and analysis of genetic networks. While perhaps not as accessible as a graphical user interface, this approach is more flexible, extensible, and amenable to automation. It can be easily combined with the large ecosystem of biological Python projects^12−15^ and uses simple programming concepts that are commonly understood by researchers from a range of disciplines.

## Results

LOICA provides a high-level genetic design abstraction using a simple and flexible object-oriented programming approach in Python. LOICA integrates models with experimental data *via* two-way communication with Flapjack, a data management and analysis tool for genetic network characterization.^13^ LOICA objects can be represented using SBOL3, enabling direct translation to part composition and DNA sequence as well as connection to repositories such as SynBioHub^16^ and the ecosystem of SBOL tools.^1−4^ All of the code, examples, and documentation are publicly available at https://github.com/RudgeLab/LOICA.

### Design Abstraction for Genetic Networks

**Figure 1:**
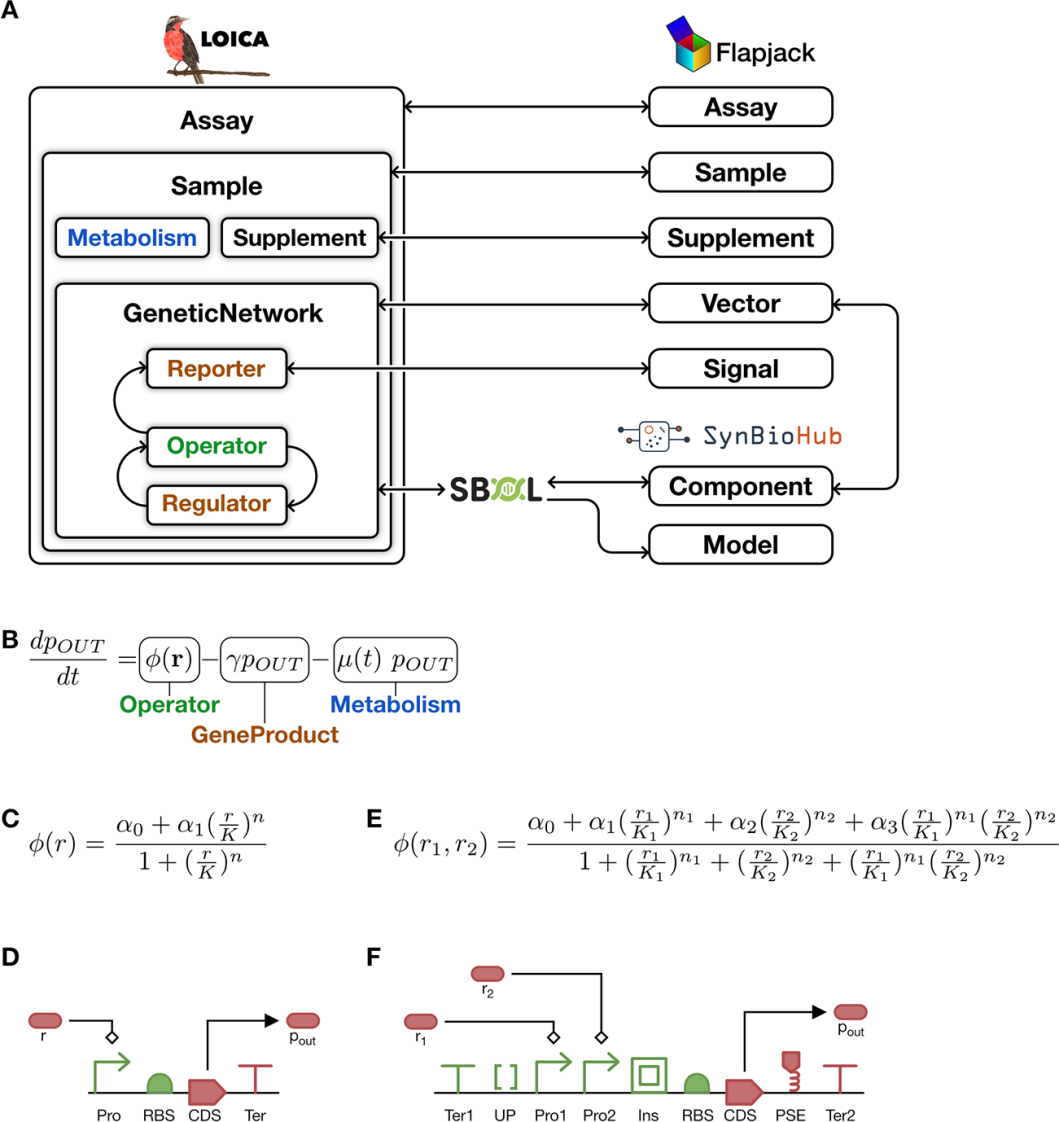
Model generation in LOICA. (A) Diagram of an *Assay* encapsulating a *Sample* that in turn encapsulates *Metabolism*, *Supplement*, and *GeneticNetwork*. In the latter, the *Operator* and *Regulator* interact to generate a model. On the right side the different interactions with the Flapjack and SBOL models are shown. (B) General mathematical model of gene expression of the *GeneProduct p*_OUT_ (*Regulator* or *Reporter*). In the *Operator*, ϕ is a transfer function that maps the concentration of the the input *r* into the *p*_OUT_ synthesis rate. In the *GeneProduct*, γ is the degradation rate of *p*_OUT_. In *Metabolism*, μ(*t*) is the instantaneous growth rate that dilutes *p*_OUT_. (C) Transfer function of a one-input *Operator*, where α_0_ and α_1_ are the nonregulated and regulated synthesis rates, respectively, *r* is the input concentration, *K* is the switching concentration, and *n* is the cooperativity degree. (D) SBOL diagram of a simple transcriptional unit that can instantiate a one-input *Operator* connected to an output *GeneProduct*. (E) Transfer function of a two-input *Operator*, where α_0_, α_1_, α_2_, and α_3_ are the nonregulated, promoter 1 regulation, promoter 2 regulation, and joint regulation synthesis rates, respectively, *r*_1_ is the input concentration of *Regulator* 1, *r*_2_ is the input concentration of *Regulator* 2, *K*_1_ is the switching concentration of promoter 1, *K*_2_ is the switching concentration of promoter 2, and *n*_1_ and *n*_2_ are the cooperativity degrees of the *Regulators* with respect to the promoters. (E) SBOL diagram of a complex transcriptional unit that can instantiate a two-input *Operator* connected to an output *GeneProduct*. SBOL diagrams were made using SBOLCanvas.^2^ The SynBioHub logo was adapted with permission from the developers^16^ and shared under a BSD 2-Clause License. Copyright 2018 SynBioHub Developers. The Flapjack logo was adapted with permission from the developers^13^ and shared under an MIT license. Copyright 2022 RudgeLab.

The basic objects in LOICA are *Operator* and *GeneProduct*, which may be either a *Regulator* or *Reporter* (Figure 1A). A *Regulator* generates a molecular species that regulates gene expression. A *Reporter* generates a molecular species that provides a measurable signal, such as a fluorescent protein. The *Operator* maps one or more *Regulator* concentrations to one or more *GeneProduct* synthesis rates. An *Operator* can be implemented in DNA as a combination of promoters and their upstream elements and downstream RBSs, and the *GeneProduct* can be a combination of CDSs, possibly a Protein Stability Element (PSE), and terminators. These objects can be represented by SBOL *Components* containing features that describe individual parts and their DNA sequences. The interactions between the *Operators* and the *Regulators* encode models for genetic network temporal dynamics, which can be simulated with ordinary differential equations (ODEs) or the stochastic simulation algorithm (SSA). The system of ODEs is thus:
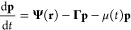
1
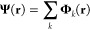
2where **p** = (*p*_0_, *p*_1_, ..., *p*_*N*–1_)^T^ is the vector of *GeneProducts*, which includes different *Regulators* (**r** = (*r*_0_, *r*_1_, ..., *r*_*M*–1_)^T^) and *Reporters* (**s** = (*s*_0_, *s*_1_, ..., *s*_*N*–*M*–1_)^T^). The nonlinear operator **Ψ** maps *Regulator* concentrations to *GeneProduct* synthesis rates. **Γ** is a diagonal matrix of *GeneProduct* degradation rates γ_*i*_, and μ(*t*) is the instantaneous growth rate of the cells. Equation 1 shows the overall system, where **Ψ** encodes the whole network and consists of a sum of individual LOICA *Operators***Φ**_*k*_ (eq 2).

In the stochastic simulation approach, these *Operators* encode the *GeneProduct* production reactions (* → *p*_*i*_) with propensities *a*_*i*_ given by the sum of *Operator* synthesis rates:
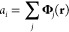
3where the sum is over all *Operators* that synthesize *GeneProducti*. The degradation rate γ_*i*_ and growth rate μ(*t*) determine the propensities *b*_*i*_ of the *GeneProduct* extinction reactions (*p*_*i*_ → *):
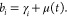
4

To make this abstract framework more concrete, Figure 1C–F shows two *Operators* currently implemented in LOICA. In the first, the output expression rate is a simple Hill function of the input *Regulator* concentration (Figure 1C). Depending on the parameters α_0_ and α_1_, this *Operator* may encode NOT logic (α_0_ > α_1_) or a Buffer (α_0_ < α_1_). The *Operator* is the set of genetic parts that regulate gene expression (Figure 1D). At its core is a promoter containing repressor or activator binding sites, such that the input *Regulator* either increases or decreases transcription and thus the gene expression rate. The second *Operator* is a two-input function (Figure 1E) that models two promoters in tandem (Figure 1F). Depending on the parameters α_0_, α_1_, α_2_, and α_3_, the *Operator* may encode a range of logic, including the NOR operation for α_0_ > α_1_, α_2_, α_3_. *Operator* instantiations may include terminators to isolate from adjacent transcription, an UP element to insulate from upstream DNA context, or an RBS and an insulator to ensure independence of the promoter and RBS function (Figure 1F).

Logical *Operators* can thus be instantiated as genetic devices that are regulated by input *Regulators* and output *GeneProduct* synthesis rates. As well as the one-input and two-input *Operators* described above, LOICA currently includes signal *Receivers* and constitutive *Sources*. LOICA currently cannot represent networks with nodes with more than two inputs, but all *Operators* can drive multiple output *GeneProducts*. However, it should be noted that LOICA can be used to define an *Operator* as any operation that maps an input *Regulator* concentration to an output synthesis rate, which may correspond to different genetic implementations than those described here. Thus, by expanding the range of *Operator* classes, in the future LOICA could be extended to represent a larger range of genetic networks.

### Model Generation and Simulation

Oscillators offer a useful dynamical system case study because they produce continuous sustained oscillations that cannot be captured by ON/OFF logic.^5,7,17−19^ We consider a genetic network based on the topology of the mammalian oscillator developed by Tigges *et al.*,^18^ consisting of positive and negative feedback loops.

**Figure 2:**
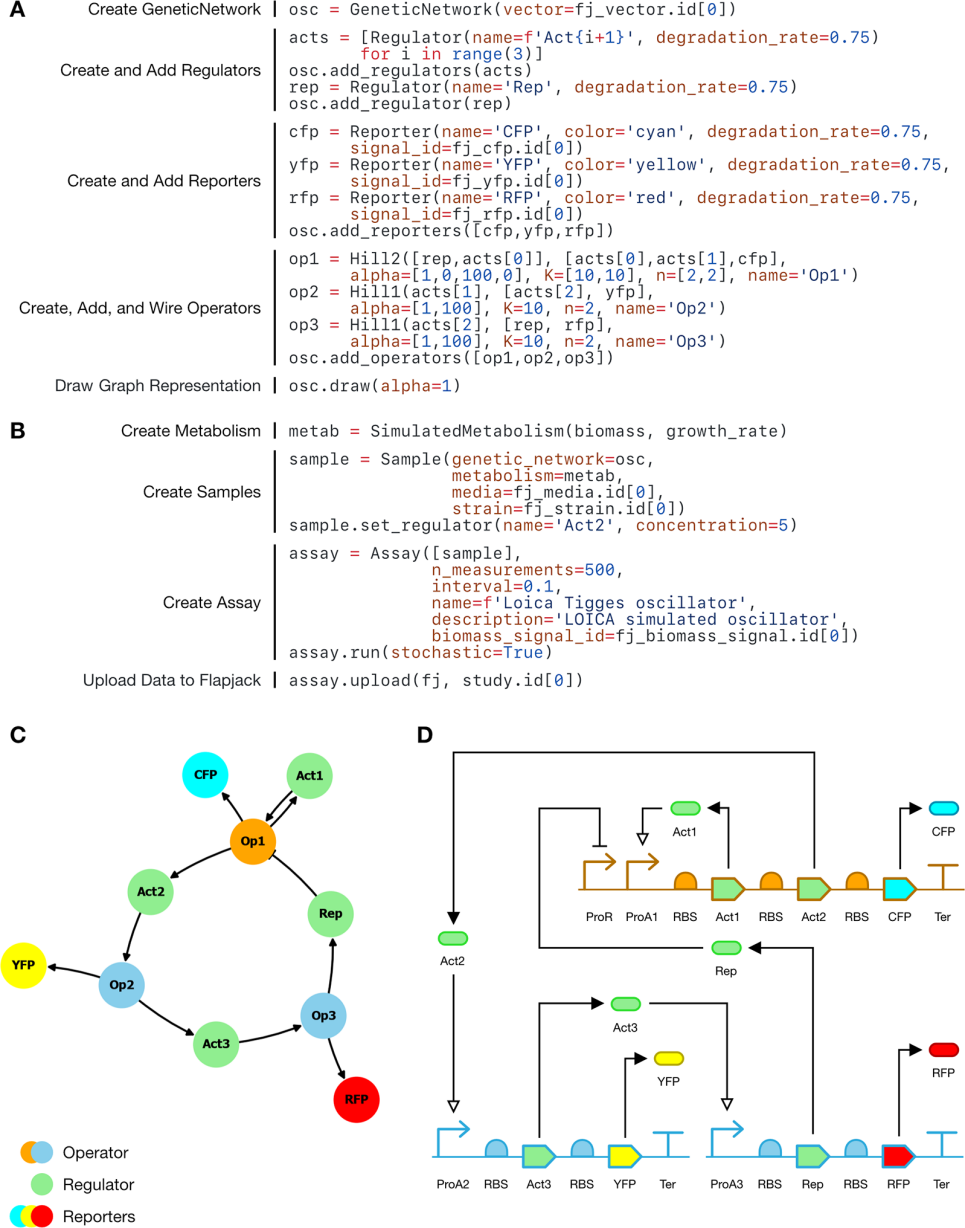
Example of oscillator design, modeling, and analysis in LOICA. (A) Python code that generates an oscillator in LOICA. *GeneticNetwork* construction is the first step, in which the user states all of the objects and their interactions. A graph representation of the model can be drawn in one function call. (B) Next, during *Assay* setup, the user initializes and runs the simulation, and the results can be uploaded to Flapjack. The two-way communication with Flapjack allows data storage and management, enables various analyses to be performed, and allows *Operators* to characterize themselves. (C, D) Comparison of graphical representations for the generated network: (C) LOICA graph output of the oscillator model and respective symbols, which demonstrates how easy it is to visualize generated networks using a higher level of abstraction; (D) SBOL representation of the generated network. It can be seen that as more *Operators* are added, the complexity for visualizing the generated network increases.

**Figure 3:**
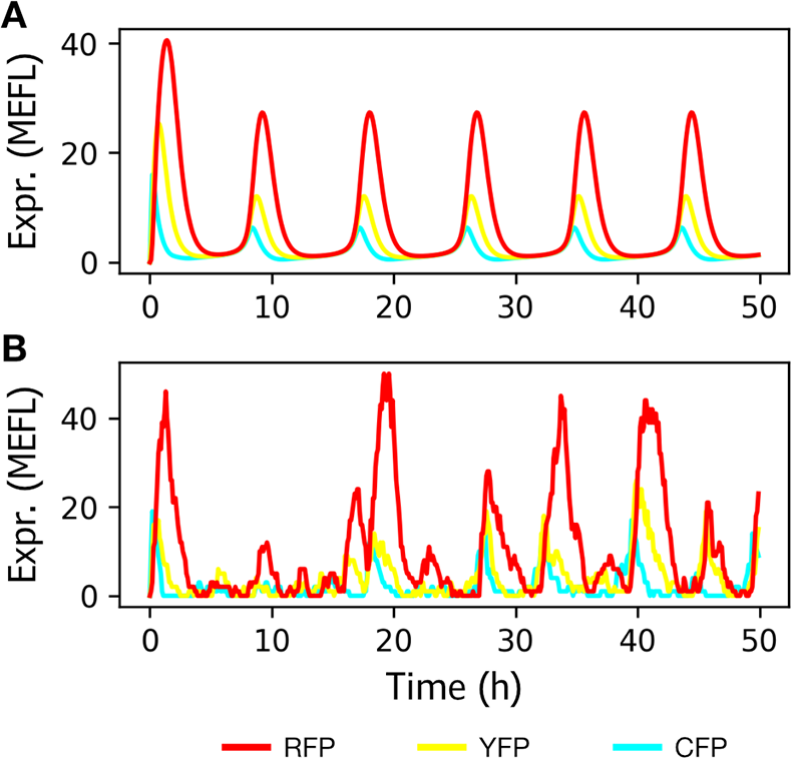
Plots of simulated data using different models. (A) Plot of simulated data from the previous network using the ODE model. (B) Plot of simulated data from the same network using the SSA model.

The design is made programmatically (Figure 2A, B) and includes three *Operators*, a two-input Hill function *Operator* (orange node in Figure 2C), which is both negatively and positively regulated, and two one-input Hill function *Operators* (blue nodes in Figure 2C), which are both activated by their respective *Regulators*. Each *Operator* also outputs a fluorescent protein *Reporter* (RFP, YFP, or CFP). The *Reporters* are linked to the Flapjack *Signal* model and together with the *Operators* and *Regulators* are incorporated into a *GeneticNetwork*, linked to the Flapjack *Vector*, which with the *Metabolism* drives the dynamics of the *Sample* (Figure 1A). The *Sample* belongs to an *Assay*, and both are connected to their corresponding Flapjack counterparts (Figure 1A). The code to create the *GeneticNetwork* model is shown in Figure 2A, and the code to define a context for modeling the genetic network is shown in Figure 2B. This approach is used to generate synthetic data using either ODEs (Figure 3A) or the SSA^20^ (Figure 3B)—contained in a LOICA *Assay*—from models that can be uploaded to Flapjack. It is then easy to access Flapjack’s genetic network characterization tools, data management, and data visualization through its Python package (pyFlapjack) or web interface (http://flapjack.rudge-lab.org).

### Encoding Designs in SBOL3

*GeneticNetwork* has a method to generate an SBOL document representation of itself. *GeneticNetwork* maps to a Component that contains representations of *Operators* and *GeneProducts* and their interactions. The combination of an *Operator* and its *GeneProducts* is represented by SubComponents of a transcriptional unit Component of type DNA with role Engineered Region. Each *GeneProduct* has a genetic production Interaction that generates a molecular species (protein or RNA). If the molecular species is a *Regulator*, then it has an inhibition or stimulation Interaction with one or more *Operators*. Whether it is an inhibition or stimulation depends on the parameters of the *Operator*. A Model is added to the SBOL document and includes the source, language, and framework. To enable the synthesis or assembly of the design, the *Operator* and *GeneProduct* Components should include a Sequence. Constraints are added to ensure correct part order.

LOICA can also generate graph representations of *GeneticNetworks*, which can be used for further analysis of their structure and for visual inspection (see Figure 2C). In comparison with the SBOL Visual representation of the same *GeneticNetwork* (Figure 2D), the LOICA representation abstracts implementation details in favor of providing a simplified design overview. Load and save functions also provide a simple way to store and exchange high-level designs.

### Operator Model Parametrization

A description of how to use LOICA to generate and analyze simulated data from models has been provided. However, another workflow scenario goes from data to model parametrization. We demonstrate this process for a two-input *Operator* using simulated data (see the example notebook https://github.com/RudgeLab/LOICA/blob/master/notebooks/Hill2.ipynb)

In order to characterize the two-input *Operator*, three auxiliary genetic networks are required. Each genetic network needs a *Reporter* as a measurable output. In this example, we used LOICA to generate simulated kinetic time-course data. The outputs must be quantified with respect to model parameters, and therefore, in an experimental setup the measurements should be properly calibrated.^21^ Two genetic networks composed of an input *Supplement* (*e.g.*, an acyl-homoserine lactone), a Receiver *Operator*, and a *Reporter* must be characterized. The Receiver *Operator* transforms the input *Supplement* concentrations into output expression rates of the *Reporter*, modeled as a Hill function. Each of these genetic networks was simulated over a range of input concentrations, and the data were uploaded to Flapjack. This allowed parametrization of the Receiver *Operators* by fitting of their dynamic models to the simulated data.

Next, the two Receiver *Operators* were composed into a genetic network to drive the *Regulator* inputs of the two-input *Operator*, which in turn drives the output *Reporter*. This genetic network was then simulated over a range of concentrations of input signals, and the data were uploaded to Flapjack. The two-input *Operator* model, combined with the parameters of the two Receivers, was then fitted to the simulated data.

The *Operator* class provides a single function that performs this parametrization process, connecting to Flapjack *via* identifiers of the appropriate genetic networks, automatically combining all of the available data.

## Discussion

The DBTL cycle is fundamental in synthetic biology, and thus, various tools have been developed to optimize the different stages. Within this cycle, modeling and characterization are essential for the design and learn stages, for which we have designed LOICA, a tool that connects these processes in an automated fashion. LOICA integrates the design, modeling, and characterization of genetic networks and their components into Python workspaces, providing a powerful and easy-to-understand high-level design abstraction for GDA that is implemented using simple object-oriented programming principles. Importantly, this programming interface does not require specialist or domain-specific knowledge but instead leverages common programming skills, making it accessible but also providing customization capabilities for advanced users.

LOICA genetic network designs are composed of objects that correspond to DNA sequences and are capable of characterizing themselves *via* links to specific experimental data in Flapjack.^13^ In this way, designs and their DNA instantiations are associated with online repositories of experimental data, which enable upload and sharing by multiple users or laboratories. This allows division of labor and reuse of experimental data that could be integrated into the workflow of distributed biofoundries. *GeneticNetworks* can automatically build SBOL3 representations of their structure that encapsulate SBOL3 Components defining LOICA objects and incorporating their part composition and DNA sequence. As well as enabling GDA, this provides an easy way to construct SBOL3 documents promoting the use of the standard and providing the capacity to export SBOL3 files, which then can be loaded to different SBOL-based tools. In this way, LOICA not only links models to part composition and DNA sequence, allowing automated assembly^22^ or synthesis, but also connects designs to DNA provenance and other metadata contained in repositories such as SynBioHub.^16^

Therefore, LOICA provides simple, easy-to-use, high-level design abstraction for modeling and characterization of genetic networks and their components, which connects to existing synthetic biology standards, tools, and repositories of experimental data to enable GDA. As with any abstraction, simplification comes at the cost of some limitations, which will be addressed in future revisions. The LOICA *Operator*–*Regulator* abstraction assumes a one-step *Regulator* synthesis model. If mRNA dynamics is important to network function, this is clearly not appropriate. Furthermore, since *Regulators* interact only with *Operators*, protein–protein or RNA–RNA interactions are not possible. These limitations may be overcome by implementing more complex models of *Operators* that track the dynamics of internal events such as mRNA production and input *Regulator*–*Regulator* interactions. Also, all *GeneProducts* synthesized by an *Operator* are currently assumed to be expressed at the same rate, which may be overcome by specifying more complex *Operator* models.

Another issue is calibration of experimental measurement data with respect to model parameters and the use of different units. Following best practice, we propose the use of Molecules of Equivalent Fluorescein (MEFL) as units for outputs using GFP or its derivatives.^21^ However, since many datasets are not calibrated, future versions of LOICA will incorporate explicit specification of units, allowing for conversion of experimental measurements to values directly comparable to model simulations.

LOICA explicitly has the *Metabolism* as a model component but currently includes limited interaction between this and the *GeneticNetwork*. It has previously been shown that gene expression is modulated by resource competition because of metabolic limitations and in turn has an effect on metabolism, including growth rates.^23−26^ These interactions are not currently included in LOICA models, but the necessary interactions between classes are present, meaning that given suitable models, the interactions between *Metabolism* and *GeneticNetwork* and their components could be encoded in a straightforward manner. Furthermore, the characterization method implemented in LOICA assumes that genetic parts are not affected by their compositional context. Various methods have been developed to reduce such effects to a minimum,^27,28^ but they cannot be discounted completely. It may be appropriate to develop a constraint-based specification of such interactions between specific parts, similar to the approach of Cello.^8^

Future work also includes parametrization of stochastic models,^29^ which will extend the existing characterization of continuous models. A major improvement will be the implementation of spatiotemporal dynamics of gene expression in multicellular populations, including a connection to CellModeller^30^ for individual-based modeling. SBOL integration will continue to be improved to leverage more features and to allow model consistency checking based on known interactions between *Operators* and *Regulators*, such that for example a known repressor cannot be encoded in a model as an activator. Ultimately, we aim to complete and automate the DBTL cycle through an open-source workflow that incorporates LOICA, Flapjack,^13^ SynBioHub,^16^ and tools powered by SBOL.^1^
